# Efficacy and Safety of Roxadustat in Chinese Hemodialysis Patients: A Systematic Review and Meta-Analysis

**DOI:** 10.3390/jcm12072450

**Published:** 2023-03-23

**Authors:** Qichen Liang, Xu Li, Qingyu Niu, Huiping Zhao, Li Zuo

**Affiliations:** Department of Nephrology, Peking University People’ s Hospital, Beijing 100044, China

**Keywords:** roxadustat, hemodialysis, anemia, meta-analysis, systematic review

## Abstract

(1) Background: Recently more and more Chinese clinical studies have been conducted to compare the efficacy and safety of roxadustat with erythropoiesis-stimulating agents (ESAs) in hemodialysis (HD) patients. We aimed to assess the efficacy and safety of roxadustat in Chinese HD patients. (2) Methods: The PubMed, Embase, the Cochrane Library, Web of Science, WanFang, China National Knowledge Infrastructure (CNKI), SinoMed, and VIP databases were searched from their inception to July 2022 for randomized controlled trials (RCTs) that compared the efficacy and safety of roxadustat to those of ESAs in treating anemia in Chinese HD patients. (3) Results: Twenty-one RCTs involving 1408 patients were enrolled. Our study showed that the improvement of hemoglobin (Hb) levels and iron metabolism were significantly higher in the roxadustat group than in the ESA group. Additionally, the total adverse events risk was significantly lower in the roxadustat group. (4) Conclusions: In this meta-analysis, we found that roxadustat was more effective and safer than ESAs in treating anemia in Chinese HD patients.

## 1. Introduction

Chronic kidney disease (CKD) is a growing global health concern, characterized by a rising incidence and prevalence rate [1]. Renal anemia, a common complication of CKD, is associated with an increased risk of morbidity and mortality, primarily due to the deficiency of endogenous erythropoietin (EPO) caused by impaired kidney function [2]. Inflammatory cytokines produced in damaged kidneys can lead to reduced production of endogenous EPO [3]. A study of the Chinese dialysis outcomes and practice patterns study (DOPPS) cohort revealed that around 60% of dialysis patients were unable to achieve the hemoglobin target of 110 g/L [4]. The current standard treatment for anemia in CKD patients is the use of recombinant human EPO or erythropoiesis-stimulating agents (ESAs) in conjunction with iron supplementation, as per the guideline [5]. While ESAs can provide immediate benefits by raising extremely low levels of hemoglobin (Hb) values and reducing the need for blood transfusions, there is evidence to suggest that ESAs increase the risk of stroke, hypertension, cardiovascular events, and death [6]. Furthermore, ESAs are required to be stored under strict cold conditions during transportation and storage, and some patients may develop resistance to ESAs. In light of these limitations, there is a need for new and effective treatments for anemia in CKD patients.

Hypoxia-inducible factors (HIFs) are essential transcription control complexes within the body’s oxygen-sensing system. These complexes consist of an α and β subunit and are precisely regulated by prolyl-hydroxyl domain (PHD) enzymes. Roxadustat, also known as FG-4592, is an oral HIF-prolyl-hydroxylase inhibitor (HIF-PHI) that mimics moderate hypoxia conditions, stabilizes the HIF-α subunit, stimulates endogenous EPO production, and improves iron metabolism [7]. However, it is important to also consider the potentially harmful effects of roxadustat, such as tumorigenesis and severe pulmonary hypertension. A growing body of research, including short-term phase 2 and 3 clinical trials, has confirmed the efficacy and safety of roxadustat in the Chinese population [8,9,10]. Currently, roxadustat is licensed for the treatment of renal anemia patients in China, and several randomized controlled trials have been conducted to compare the efficacy and safety of roxadustat versus ESAs in treating Chinese hemodialysis (HD) patients.

Given the importance of providing robust evidence for the clinical application of roxadustat, this meta-analysis was conducted to assess the efficacy and safety of roxadustat in Chinese HD patients. The results of this meta-analysis will provide valuable information for the clinical use of roxadustat in treating renal anemia in Chinese HD patients.

## 2. Materials and Methods

The present study was conducted in accordance with the Preferred Reporting Items for Systematic Reviews and Meta-Analyses (PRISMA) Statement guideline [11] and registered in the PROSPERO database (Registration number: CRD42022347307). The PRISMA checklist, which outlines the requirements for reporting systematic reviews and meta-analyses, is listed in Table S1 of the study.

### 2.1. Search Strategy

In order to gather relevant information for this study, we carried out a comprehensive search of several databases, including Pubmed, Embase, the Cochrane Library, Web of science, WanFang, China National Knowledge Infrastructure, SinoMed, and VIP databases. The search was performed from the inception of each database up until 19 July 2022. The search was conducted using MeSH terms and a combination of free text words that included “roxadustat”, “HIF-PHI”, “FG-4592”, “anemia”, “anemias”, “Chinese”, and “China” .ClinicalTrials.gov and the references cited in selected papers and other previous reviews were also searched.

### 2.2. Inclusion Criteria

In order to be included in this study, the trials had to meet the following criteria: (1) they had to be randomized controlled trials (RCTs) performed on HD patients with anemia; (2) the experimental group had to be exposed to roxadustat, while the controls were treated with ESAs; (3) the studies had to have a follow-up duration of at least 8 weeks; (4) at least one of the following outcomes had to be reported: change in Hb level, change in transferrin level, change in hepcidin level, change in transferrin saturation (TSAT), change in ferritin, change in total iron-binding capacity (TIBC), change in serum iron (SI), and adverse events (AEs). 

### 2.3. Data Extraction and Quality Assessment

To extract the relevant information from the studies that met the inclusion criteria, the following details were recorded: the name of the study, publication year, authors, study type, number of patients, age, gender, duration of dialysis, doses used in the trial, and reported outcomes (Hb, TSAT, ferritin, transferrin, hepcidin, TIBC, SI, and AEs). Two reviewers (Q.L. and X.L.) independently conducted a quality assessment of the included studies using the Cochrane Collaboration’s Risk of Bias tool [12]. The assessment was performed to evaluate the risk of bias in the studies, taking into account factors, such as random sequence generation, allocation concealment, blinding of participants and health care personnel, blinding of outcome assessment, incomplete outcome data, selective reporting, and other biases. In the case of disagreements, a third reviewer (L.Z.) was consulted to resolve the issue.

### 2.4. Statistical Analysis

The statistical analysis for this study was carried out using a combination of Review Manager software (RevMan, Version 5.4.1, The Cochrane Collaboration, 2020) and Stata 14.0 (StataCorp LP, College Station, TX, USA). Two researchers performed the analysis independently in order to avoid entry errors. The mean objective of the analysis was to compare the efficacy of roxadustat with that of ESAs in the treatment of anemia in Chinese HD patients. The study employed two statistical measures, the mean difference (MD) and risk ratio (RR) with 95% confidence interval (CI), to calculate the difference between the roxadustat and ESA groups. The Cochrane Q test was used to evaluate the heterogeneity of the results, which refers to the degree of variability of the results across different studies. Based on the results of the Cochrane Q test, either a fixed-effect model or a random-effect model was applied. The fixed-effect model was used if the I^2^ statistic was less than 50% and the *p* value was greater than 0.1. A sensitivity analysis was conducted to assess the robustness of the results. The analysis involved excluding each study one by one and recalculating the statistical measures. Begg’s and Egger’s tests were also conducted to examine the potential for publication bias, which refers to the phenomenon of studies with positive results being more likely to be published than studies with negative results. All tests were two-tailed, and a *p* value less than 0.05 was considered statistically significant.

## 3. Results

### 3.1. Literature Selection and Study Characteristics

**Figure 1:**
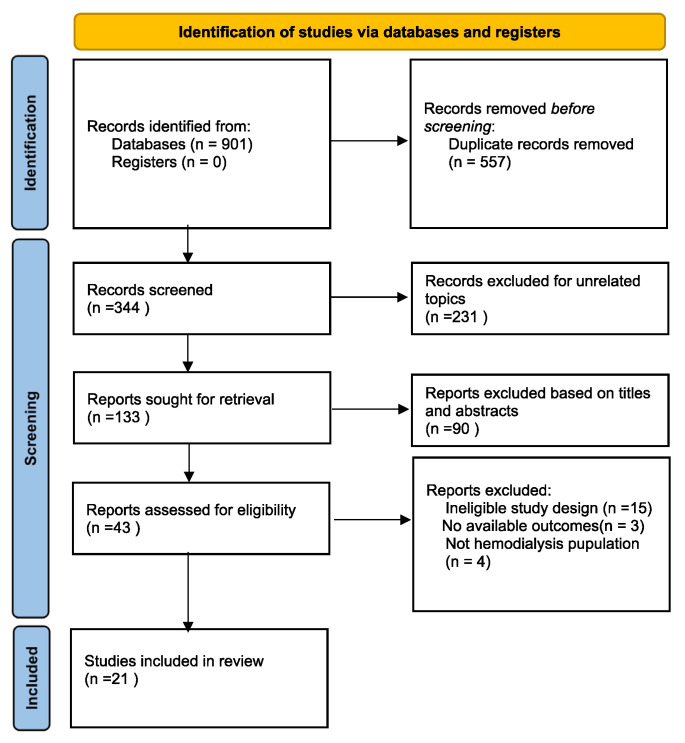
PRISMA 2020 Flow diagram of the identification process for eligible studies.

The entire search strategy is shown in Figure 1. Initially, 901 records were found based on our search strategy. Once duplicates had been removed, 344 abstracts were screened. Based on the titles and abstracts, 133 publications were selected for full-text review. Finally, a total of 21 studies with a total of 1408 participants were included in this meta-analysis. Characteristics of the 21 studies included are presented in Table 1. All 21 studies were ESA controlled.

### 3.2. Risk of Bias Assessment

**Figure 2:**
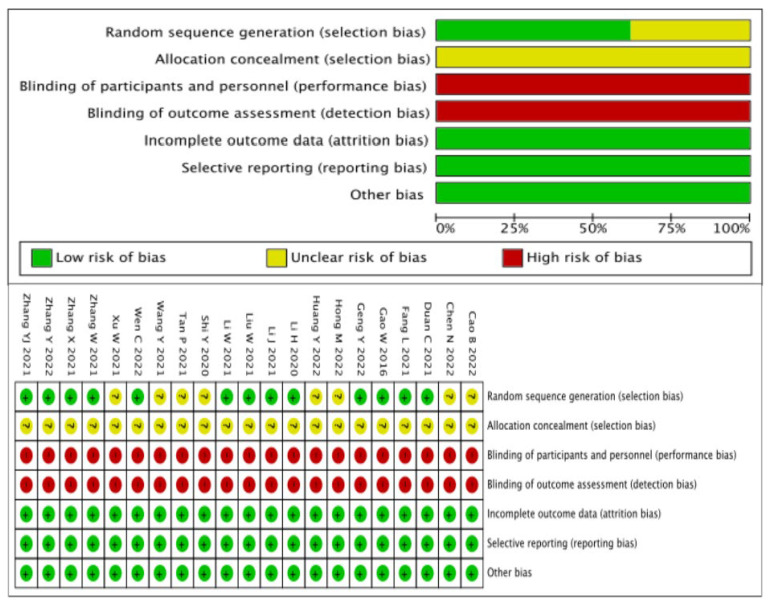
Risk of bias assessment of the studies [13,14,15,16,17,18,19,20,21,22,23,24,25,26,27,28,29,30,31,32]. “+”, low risk of bias; “−“, high risk of bias; “?”, unclear risk of bias.

The risk of bias assessment is presented in Figure 2. The Cochrane Collaboration’s Risk of Bias tool [12] was used to evaluate the risk of bias. The quality of all studies was acceptable. Randomized sequence generation were reported in twelve studies with details. All the studies were open-label studies due to the different usage of the two interventions, which may lead to a high risk of blinding biases.

### 3.3. Efficacy

#### 3.3.1. Change in Hb Levels from Baseline (ΔHb)

**Figure 3:**
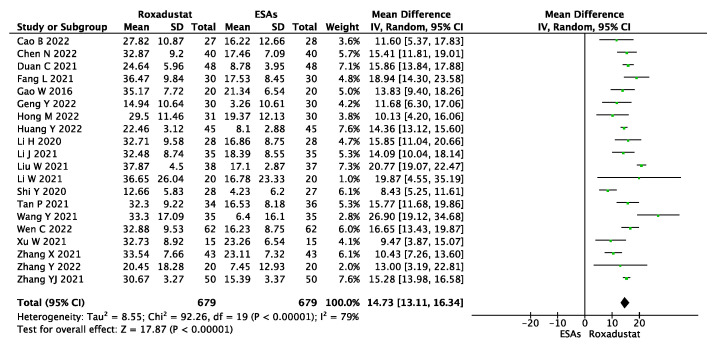
Forest plot for the outcome change in Hb level (ΔHb).

The change in Hb levels, which is the main outcome in renal anemia therapy, was used to access the efficacy of roxadustat. A total of twenty studies reported ΔHb, including 1358 patients. Using a random-effect model, the pooled results showed that ΔHb was significantly higher in the roxadustat group than in the ESA group (WMD = 14.73, 95% CI 13.11~16.34, *p* < 0.00001, I^2^ = 79%), as shown in Figure 3.

#### 3.3.2. Change in Transferrin Levels from Baseline (Δtransferrin)

**Figure 4:**
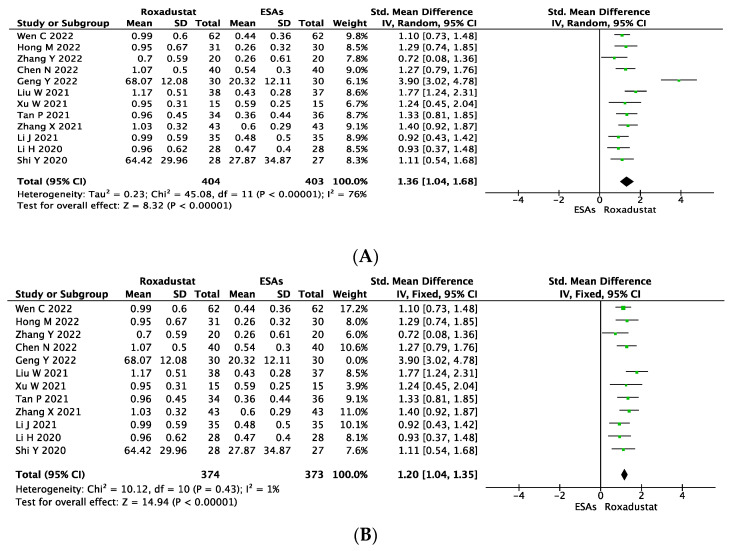
Forest plot for the outcome change in transferrin levels (Δtransterrin) (**A**). Sensitivity analysis was conducted by excluding each study one by one to find out the origin of heterogeneity. After excluding Geng et al.’s study, heterogeneity disappeared (I^2^ = 1%) (**B**).

The change in transferrin levels was reported in twelve studies with 404 subjects in the roxadustat group and 403 subjects in the ESA group (SMD = 1.36, 95% CI 1.04~1.68, *p* < 0.00001, I^2^ = 76%, Figure 4A). After conducting a sensitivity analysis by excluding Geng et al.’s study [19], the I^2^ fell to 1%, while the Δtransferrin level was significantly higher for roxadustat than ESAs (SMD = 1.20, 95% CI 1.04~1.35, *p* < 0.00001, I^2^ = 1%, Figure 4B).

#### 3.3.3. Change in Hepcidin Levels from Baseline (Δhepcidin)

**Figure 5:**
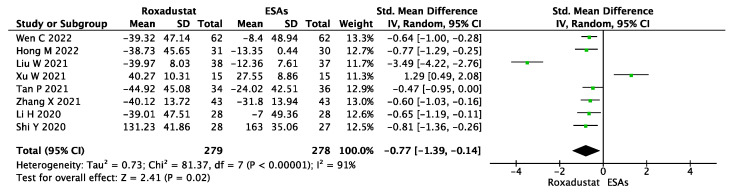
Forest plot for the outcome change in hepcidin levels (Δhepcidin).

The Δhepcidin levels were reported in eight studies compared for roxadustat versus ESAs. The pooled results using a random-effect model demonstrated that Δhepcidin was significantly lower in the roxadustat group (SMD = −0.77, 95% CI −1.39~−0.14, *p* = 0.02, I^2^ = 91%), as shown in Figure 5.

#### 3.3.4. Change in TSAT% from Baseline (ΔTSAT%)

**Figure 6:**
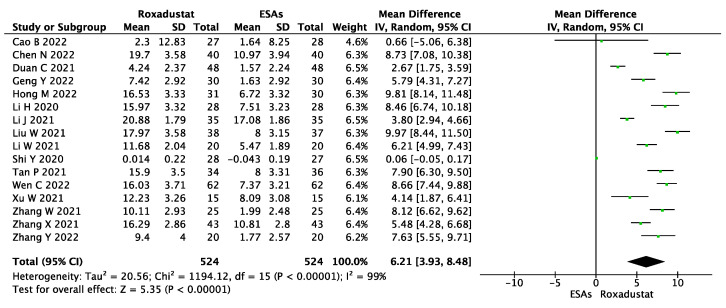
Forest plot for the outcome change in TSAT% levels (ΔTSAT%).

The ΔTSAT% was examined in sixteen studies, which included 1048 patients. We pooled the results using a random-effect model and found that, compared with that in the ESA group, the roxadustat group had a much higher ΔTSAT% level (WMD = 6.21, 95% CI 3.93~8.48, *p* < 0.00001, I^2^ = 99%), as shown in Figure 6.

#### 3.3.5. Change in Ferritin Levels from Baseline (Δferritin)

**Figure 7:**
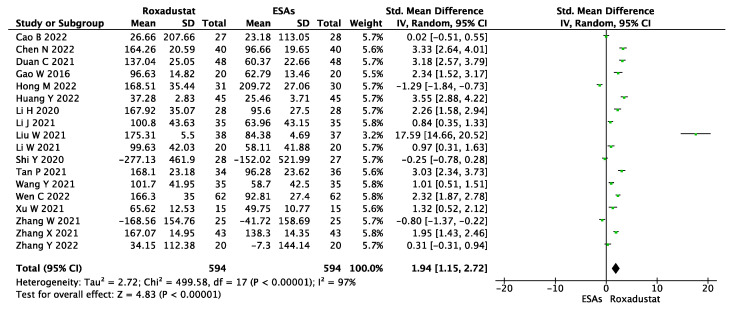
Forest plot for the outcome change in ferritin levels (Δferritin).

The change in ferritin levels was available in eighteen studies with 1188 patients, as shown in Figure 7. Using a random-effect model, the pooled results showed that roxadustat significantly increased the ferritin levels compared to those with ESAs (SMD = 1.94, 95% CI 1.15~2.72, *p* < 0.00001, I^2^ = 97%).

#### 3.3.6. Change in TIBC Levels from Baseline (ΔTIBC)

**Figure 8:**
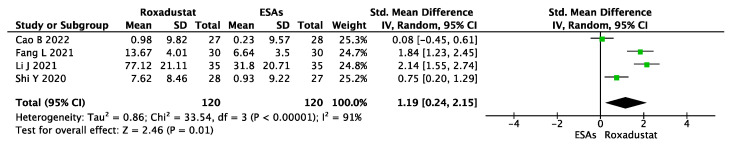
Forest plot for the outcome change in TIBC levels (ΔTIBC).

The change in TIBC levels was reported in four studies with 240 patients. The pooled results with a random-effect model showed that ΔTIBC was significantly increased in the roxadustat group compared to that with ESAs (SMD = 1.19, 95% CI 0.24~2.15, *p* = 0.01, I^2^ = 91%), as shown in Figure 8.

#### 3.3.7. Change in SI Levels from Baseline (ΔSI)

**Figure 9:**
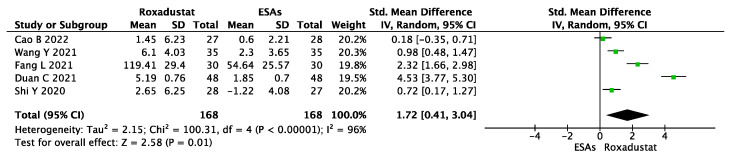
Forest plot for the outcome change in SI levels (ΔSI).

When compared to that in the ESA group, roxadustat resulted in an increase in the SI level (SMD = 1.72, 95% CI 0.41~3.04, *p* = 0.01, I^2^ = 96%), as shown in Figure 9.

### 3.4. Safety (AEs)

**Figure 10:**
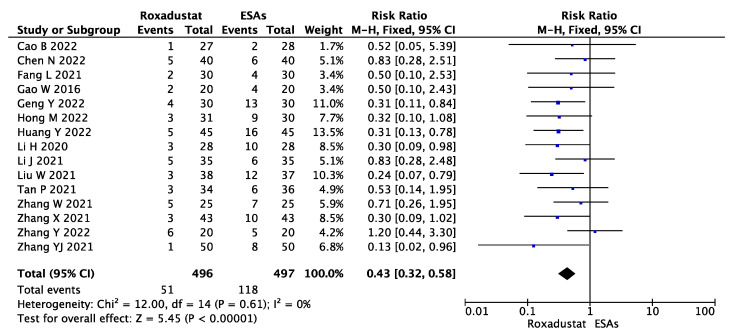
Forest plot for AEs.

We accessed the safety of roxadustat by analyzing the rate of adverse events data, which were reported in fifteen studies. Among 496 patients in the roxadustat group and 497 patients in ESA group, our meta-analysis results revealed that the roxadustat group had a significantly lower risk of AEs than the ESA group (RR = 0.43, 95% CI 0.32~0.58, *p* < 0.00001, I^2^ = 0%), as shown in Figure 10.

### 3.5. Publication Bias

Begg’s and Egger’s tests were utilized to estimate publication bias (Table 2). Our meta-analysis indicated no publication bias except for TSAT (*p*_Egger_ < 0.05) and ferritin (*p*_Egger_ = 0.016), where there may have been publication bias. Since the included studies were less than ten, we did not perform publication bias tests on TIBC and SI.

## 4. Discussion

Our meta-analysis, which was based on 21 studies and a total of 1408 participants, aimed to compare the efficacy and safety of roxadustat versus ESAs in Chinese HD patients. Our findings showed that, compared to those with ESAs, roxadustat significantly increased Hb levels, transferrin, TSAT, and ferritin levels and decreased hepcidin levels. Additionally, roxadustat was associated with a lower risk of AEs compared to that with ESAs.

Our meta-analysis confirmed the efficacy of roxadustat in increasing Hb and improving renal anemia in Chinese HD patients, which is consistent with several meta-analyses [34,35,36]. Roxadustat, also known as FG–4592, is an oral second-generation HIF-PHI. HIF is a heterodimer consisting of two subunits: oxygen-responsive HIF α, and constitutive HIF β. HIF-α has three isoforms (HIF 1α, HIF 2α, and HIF 3α), and HIF-2α plays a crucial role in normal erythropoietin production. When HIF-2α combines with HIF-β, it binds to the hypoxia response element (HRE) and induces the expression of target genes [37]. The stability of HIF-α is regulated by 2–oxoglutarate–dependent oxygenases, also known as PHD enzymes, of which PHD2 is the predominant regulator. Under normoxic conditions, HIF-2α is hydroxylated by PHD and consequently recognized by the von Hippel Lindau (VHL) E3 ubiquitin ligase, resulting in ubiquitination and proteasomal degradation. In contrast, hypoxia inhibits the hydroxylation and thus degradation of HIF-2α, which is due to PHD needing molecular oxygen to hydroxylate HIF-2α. HIF-PHIs mimic the body’s exposure to moderate hypoxia conditions, leading to increased HIF-2α activity and hypoxia-induced gene expression, including erythropoietin. In addition, HIF-PHIs can also stimulate the synthesis of endogenous EPO in peritubular interstitial fibroblasts in injured kidneys. Renal EPO-producing cells (REPs) transform into myofibroblast-like cells and lose their capability to produce EPO during chronic nephropathies. Souma et al. revealed that the oxygen-sensing system is impaired in injured kidneys and leads to insufficient HIF responses [38]. By inhibiting PHDs, HIF signaling is augmented, resulting in the reactivation of EPO synthesis. Several randomized clinical trials have confirmed the dose-related increases in Hb levels [10,39,40]. Of note, this response is independent from the baseline C-reactive protein levels, which indicated that roxadustat may overcome the inflammatory status in CKD patients.

Our study found that roxadustat improved iron parameters by reducing the levels of hepcidin and increasing TIBC and transferrin. This finding is supported by the results of a study by Provenzano et al. [41], which showed that after 16 weeks of treatment, hepcidin levels were decreased by 16.9% (*p* = 0.004) and TIBC was increased by 15.3% (*p* < 0.001). However, the decline in TSAT and ferritin levels in Provenzano et al.’s study differs from our results. This discrepancy may be attributed to the fact that our study population consisted of HD patients, and increasing ferritin levels may be associated with the inflammatory status in HD patients. Another study by Provenzano et al. revealed that iron metabolism parameters did not change significantly from baseline in HD patients [42]. A recent updated meta-analysis also showed that, SI, TIBC, ferritin, and TSAT were increased after roxadustat treatment in dialysis-dependent (DD) patients, which supports our findings [43]. It is worth mentioning that to date, no clinical studies have been conducted to evaluate the efficacy of roxadustat in iron-deficient patients undergoing HD yet. CKD patients often have abnormally elevated hepcidin levels due to chronic inflammation and declining renal clearance, leading to functional iron deficiency (FID). Hepcidin is a kind of peptide hormone that mainly originates from the liver and is responsible for maintaining iron homeostasis. It exerts an adverse effect on erythropoiesis by combining with ferroportin (FPN), by inducing internalization and the destruction of FPN [44]. HIF-PHIs, such as roxadustat, are capable of regulating the expression of duodenal cytochrome b (Dcytb) and divalent metal transporter 1 (DMT1) to improve intestinal iron absorption [45]. Furthermore, HIF-PHIs are able to upregulate transferrin and transferrin receptor 1 so as to improve iron transport. The potential mechanism of the effect of HIF-PHIs on the hepcidin level is still unclear. Some studies revealed that HIF-PHIs reduce hepcidin levels by increasing erythropoiesis [46,47,48], while a recent study described a direct effect [49]. 

Our study also demonstrated that roxadustat was associated with a lower risk of AEs compared to that with ESAs. Several studies have linked high-dose ESAs to an increased risk of hypertension, cardiovascular events, and mortality [37]. Roxadustat has shown potential benefits in protecting organs from ischemic injury, including stroke and myocardial infarction [7]. In comparison to traditional ESAs that are exposed to high doses, roxadustat stimulates endogenous EPO at physiologic or slightly higher levels. Preclinical experiments indicated that HIF-PHIs showed hypotensive effects during the treatment of anemia [50], while clinical trials found the opposite results. Plenty of clinical trials demonstrated that roxadustat treatment significantly reduced low-density lipoprotein (LDL) cholesterol levels due to the increased degradation of 3-hydroxy-3-methylglutaryl (HMG) -CoA reductase, mediated by HIF [51,52]. An improvement of metabolic disorders, such as reducing glucose levels and insulin resistance, was also reported [53]. However, potential disadvantages of roxadustat should be taken seriously, such as diarrhea, vomiting, peripheral edema, headache, fatigue, and hyperkalemia. The most significant disadvantage is the tumor-promoting effects. The hypoxia condition mimicked by roxadustat may cause the overexpression of HIF, resulting in the risk of tumor angiogenesis. Thus, patients with cancer should be cautious in using roxadustat and other HIF-PHIs. HIF activation may also cause severe pulmonary arterial hypertension (PAH), which was demonstrated in a mouse model [54]. However, few clinical studies have been conducted to evaluate the safety of roxadustat in PAH patients because of the rarity of the disease. It is worth noting that a meta-analysis, including 30 RCTs, revealed that roxadustat may be associated with an increased risk of thromboembolic events compared to that with ESAs or placebo [55]. In contrast, an animal experiment indicated that roxadustat has no significant effect on platelet production and thrombosis formation [56]. Since HIF activation may induce the expression of a wide range of genes, some potential off-target effects of PHD inhibitors, such as reducing complement C1q levels and progression in polycystic kidney disease, should also be taken into consideration.

There are many potential beneficial effects of HIF-PHIs other than treating renal anemia. The MATTERHORN [57] study is a phase 3, randomized, double-blind study, which indicated that roxadustat treatment is effective in patients with low-risk myelodysplastic syndrome (MDS) by reducing transfusions. Preclinical investigations indicated the protective effects of HIF-PHIs on acute kidney injury (AKI). Nevertheless, since these experiments used HIF-PHIs prior to AKI, the post-injury therapeutic effect is still unknown. The recent cases reported the suppressive effect of roxadustat on thyroid-stimulating hormone (TSH) in HD patients [58,59]. The neuroprotective effect of HIF-PHIs was also discovered in animal models. Li et al. [60] found that roxadustat showed therapeutic potential in models of Parkinson’s disease (PD) by improving the mitochondrial function under oxidative stress. Surprisingly, roxadustat may also serve as a potential treatment for COVID-19 (the epidemic disease caused by the SARS-CoV-2 virus), as it is capable of inhibiting SARS-CoV-2 RNA entry and replication in lung epithelial cells [61].

There are plenty of advantages of roxadustat application compared to ESAs. Firstly, roxadustat can be administered orally, increasing the compliance of patients and eliminating the risk of patients developing antibodies to erythropoietin associated with ESA injection. Secondly, roxadustat achieves a significant anti-anemia effect in inflamed individuals resistant to ESAs. Zhou et al. [62] reported 32 EPO hypo-responsiveness patients who were transferred from ESAs to roxadustat and found that 15 cases met the Hb target. Thirdly, roxadustat can be stored at room temperature, which is easier to store than ESAs. Fourthly, the intermittent dosing schedule of roxadustat induces a lower increase in the blood levels of EPO than exogenous EPO dosing. 

Our meta-analysis is the first study that focuses exclusively on the efficacy and safety of roxadustat in Chinese HD patients. This is a valuable contribution to the literature, as previous meta-analyses have included both dialysis-dependent (DD) and non-dialysis-dependent (NDD) patients, which can introduce bias and heterogeneity into the results. By focusing solely on Chinese HD patients, our study minimizes the potential impact of these factors and provides a clearer picture of the effects of roxadustat in this patient population. Furthermore, by limiting the study to trials conducted by clinical researchers or nephrologists only, we can be confident that the results are more robust and accurate, as these trials were conducted by experts in the field.

Our meta-analysis had some limitations that should be taken into consideration. Firstly, the duration of the included studies was only between 8 weeks to 24 weeks, which is relatively short. As a result, the long-term therapeutic effect of roxadustat and the incidence of adverse events remain unclear. Further studies with longer treatment durations are necessary to determine the long-term efficacy and safety of roxadustat. Secondly, the heterogeneity among the included studies was still high, which may be due to differences in baseline Hb levels, roxadustat dosages, treatment durations, and iron supplementation. These factors may have influenced the results of our meta-analysis, and further studies controlling for these factors are needed to clarify the efficacy and safety of roxadustat. Thirdly, most of the included studies lacked reporting on random sequence generation and allocation concealment, which could have introduced some bias into the results. Future studies should include appropriate measures to reduce the risk of bias, such as using proper randomization methods and concealing allocation. In conclusion, while our meta-analysis provides valuable insights into the efficacy and safety of roxadustat in Chinese HD patients, additional high-quality studies with longer treatment durations and better control for potential confounding factors are needed to further confirm our findings.

## 5. Conclusions

In this meta-analysis, we found that roxadustat is more effective in increasing Hb levels and improving iron metabolisms than ESAs. It is also safer than ESAs in treating renal anemia in Chinese HD patients. It may be a good alternative treatment for anemia patients undergoing HD. However, the long-term safety and efficacy of roxadustat in HD patients remain to be investigated. Long-term, high-quality studies, and large-sample size studies are needed.

## Figures and Tables

**Table 1:** Basic characteristics of included studies.

Study/Year	Study Arms	Sample Size (%Men)	Age (Years) in Experimental Group	Age (Years) in Control Group	Type of Patients	Follow-Up (Weeks)
Cao B 2022 [13]	Roxadustat/ESAs	55 (47.3)	57.36 ± 7.75	58.94 ± 9.11	HD	12
Wen C 2022 [14]	Roxadustat/ESAs	124 (53.2)	41.36 ± 8.92	43.43 ± 10.15	HD	12
Hong M 2022 [15]	Roxadustat/ESAs	61 (60.7)	51.00 ± 2.90	51.40 ± 3.00	HD	8
Zhang Y 2022 [16]	Roxadustat/ESAs	40	58.10 ± 13.87	53.05 ± 14.85	HD	12
Chen N 2022 [17]	Roxadustat/ESAs	80 (47.5)	53.40 ± 3.07	53.33 ± 3.11	HD	12
Huang Y 2022 [18]	Roxadustat/ESAs	90 (58.9)	62.92 ± 4.88	61.32 ± 4.55	HD	12
Geng Y 2022 [19]	Roxadustat/ESAs	60 (58.3)	42.63 ± 3.18	42.61 ± 3.16	HD	8
Li W 2021 [20]	Roxadustat/ESAs	40 (57.5)	54.29 ± 7.11	54.63 ± 7.14	HD	8
Liu W 2021 [21]	Roxadustat/ESAs	75 (50.7)	48.91 ± 3.12	48.96 ± 3.18	HD	12
Xu W 2021 [22]	Roxadustat/ESAs	30 (56.7)	51.38 ± 9.26	51.79 ± 9.31	HD	12
Zhang YJ 2021 [23]	Roxadustat/ESAs	100 (63.0)	45.40 ± 1.11	45.00 ± 1.12	HD	12
Zhang W 2021 [24]	Roxadustat/ESAs	50 (54.0)	42.57 ± 3.42	43.53 ± 3.27	HD	12
Wang Y 2021 [25]	Roxadustat/ESAs	70	NA	NA	HD	8
Tan P 2021 [26]	Roxadustat/ESAs	70 (51.4)	45.60 ± 7.10	47.60 ± 6.70	HD	12
Fang L 2021 [27]	Roxadustat/ESAs	60	NA	NA	HD	12
Zhang X 2021 [28]	Roxadustat/ESAs	86	NA	NA	HD	12
Duan C 2021 [29]	Roxadustat/ESAs	96 (62.5)	61.63 ± 5.78	61.59 ± 5.73	HD	8
Li J 2021 [30]	Roxadustat/ESAs	70 (51.4)	43.29 ± 4.05	44.96 ± 3.82	HD	8
Li H2020 [31]	Roxadustat/ESAs	56 (55.4)	44.13 ± 3.69	43.95 ± 3.61	HD	12
Shi Y 2020 [32]	Roxadustat/ESAs	55	53.43 ± 14.74	56.33 ± 10.50	HD	24
Gao W 2016 [33]	Roxadustat/ESAs	40 (57.5)	48.25 ± 1.61	48.23 ± 1.56	HD	12

Participant characteristics are presented as the mean ± SD. ESAs, erythropoiesis-stimulating agents; HD, hemodialysis; NA, not available.

**Table 2:** *p* value of Begg’s and Egger’s tests.

Test	Hb	Transferrin	Hepcidin	TSAT	Ferritin	AEs
Begg’s	0.820	0.533	0.536	1.000	0.081	0.692
Egger’s	0.500	0.919	0.743	<0.05	0.016	0.418

Hb, hemoglobin; TSAT, transferrin saturation; AEs, adverse events.

## Data Availability

Please see the original included articles.

## Supplementary Materials

The following supporting information can be downloaded at: https://www.mdpi.com/article/10.3390/jcm12072450/s1. Table S1: PRISMA 2020 checklist.
